# Update of the keratin gene family: evolution, tissue-specific expression patterns, and relevance to clinical disorders

**DOI:** 10.1186/s40246-021-00374-9

**Published:** 2022-01-06

**Authors:** Minh Ho, Brian Thompson, Jeffrey Nicholas Fisk, Daniel W. Nebert, Elspeth A. Bruford, Vasilis Vasiliou, Christopher G. Bunick

**Affiliations:** 1 Department of Dermatology, Yale University, 333 Cedar St., LCI 501, PO Box 208059, New Haven, CT 06520-8059 USA; 2 Department of Environmental Health Sciences, Yale School of Public Health, New Haven, CT 06511 USA; 3 Program of Computational Biology and Bioinformatics, Yale University, New Haven, CT 06511 USA; 4 Departments of Pediatrics and Molecular and Developmental Biology, Cincinnati Children’s Research Center, Cincinnati, OH 45229 USA; 5 Department of Environmental Health and Center for Environmental Genetics, University of Cincinnati College of Medicine, Cincinnati, OH 45267 USA; 6 HUGO Gene Nomenclature Committee (HGNC), EMBL-EBI, Wellcome Genome Campus, Hinxton, Cambridge, CB10 1SD UK; 7 Department of Haematology, University of Cambridge, Cambridge, CB2 0XY UK; 8 Department of Molecular Biophysics and Biochemistry, Yale University, New Haven, CT 06520 USA

**Keywords:** Keratin, Intermediate filament, Evolutionary blooms, Gene expression, Gene duplications, Synteny, Markov-chain Monte Carlo (MCMC), MrBayes program to estimate phylogeny

## Abstract

Intermediate filament (IntFil) genes arose during early metazoan evolution, to provide mechanical support for plasma membranes contacting/interacting with other cells and the extracellular matrix. Keratin genes comprise the largest subset of IntFil genes. Whereas the first keratin gene appeared in sponge, and three genes in arthropods, more rapid increases in keratin genes occurred in lungfish and amphibian genomes, concomitant with land animal-sea animal divergence (~ 440 to 410 million years ago). Human, mouse and zebrafish genomes contain 18, 17 and 24 non-keratin IntFil genes, respectively. Human has 27 of 28 type I “acidic” keratin genes clustered at chromosome (Chr) 17q21.2, and all 26 type II “basic” keratin genes clustered at Chr 12q13.13. Mouse has 27 of 28 type I keratin genes clustered on Chr 11, and all 26 type II clustered on Chr 15. Zebrafish has 18 type I keratin genes scattered on five chromosomes, and 3 type II keratin genes on two chromosomes. Types I and II keratin clusters—reflecting evolutionary blooms of keratin genes along one chromosomal segment—are found in all land animal genomes examined, but not fishes; such rapid gene expansions likely reflect sudden requirements for many novel paralogous proteins having divergent functions to enhance species survival following sea-to-land transition. Using data from the Genotype-Tissue Expression (GTEx) project, tissue-specific keratin expression throughout the human body was reconstructed. Clustering of gene expression patterns revealed similarities in tissue-specific expression patterns for previously described “keratin pairs” (i.e., KRT1/KRT10, KRT8/KRT18, KRT5/KRT14, KRT6/KRT16 and KRT6/KRT17 proteins). The ClinVar database currently lists 26 human disease-causing variants within the various domains of keratin proteins.

**Supplementary Information:**

The online version contains supplementary material available at 10.1186/s40246-021-00374-9.

## Background

### Intermediate filaments: historical background

By end of the Cambrian explosion (~ 500 million years ago), intermediate filament (IntFil) genes had become well established in the *Animalia* Kingdom and began expanding rapidly, encoding novel proteins that were necessary for species survival among metazoans. These IntFil genes played dynamic roles in cell integrity and structural scaffolding—more specifically, to provide mechanical support for plasma membranes where they come into contact with other cells and with the extracellular matrix.

The scientific discovery of IntFils coincided with the birth of structural biology, *e.g.,* William Astbury [1] detected hair and wool diffraction patterns on X-ray photographs in 1931. Building off Linus Pauling’s discovery in the 1950s that a protein’s secondary structure consists of α-helices and β-sheets, Francis Crick elucidated that hair keratin’s X-ray diffraction patterns were consistent with coiled-coil α-helices [2].

IntFils originally were mistaken as part of the “myofibrils group,” until Howard Holtzer performed careful electron microscopy experiments and determined that IntFils were 10-nm thick in diameter, as compared with myofibrils (15-nm diameter); hence, the name “intermediate-sized filaments” [3]. In the following years, techniques for isolating and denaturing/reassembling IntFils were fine-tuned for better observation via electron microscopy [4, 5]. These improved techniques have facilitated a better understanding of IntFil protein structure and the role of IntFils in many human diseases.

By the early 1990s IntFils had been categorized into six classes (i.e., types I, II, III, IV, V & VI), based on tissue-specific expression patterns, identified by immunofluorescence [6]. Type I “acidic” keratin and type II “basic” keratin expressions are highest in epithelial cells, hair, and nails [7]. Type III IntFil proteins—which include vimentin, desmin, peripherin and glial fibrillary acidic protein—are expressed in mesenchymal, myogenic, neuronal, and glial cells, respectively [8–11]. Expression of type IV neurofilaments is limited to neuronal cells [12]. Type V lamins are expressed in all cells, where they function mostly in the nuclear lamina [13]. Type VI filensin and phakinin were discovered most recently; their expression appears to be limited to the lens of the eye [14, 15].

The advent of high-throughput genomic-sequencing technologies has greatly facilitated identification of new IntFil group members [7]. Unfortunately, identification of these new IntFil group members, and in particular the keratin genes, has greatly complicated nomenclature of these genes and has led to substantial confusion. Thus, in 2005, a standardized nomenclature system (https://www.genenames.org/) was established for keratin genes [7]. Due to high similarity in sequence, and vast variations in expression and functionalities among different cell types, functional characterization of some IntFil members continues to be poorly understood.

### IntFil proteins: structure and assembly

The structural domain organization of IntFils is very similar—consisting of a highly conserved α-helix central rod domain, flanked by non-helical amino acids at both the NH_2_-terminus (head) and COOH-terminus (tail) domains. Importantly, the core α-helix is constructed in a repeating heptad pattern of amino acids [e.g., (*abcdefg*)_n_] with apolar residues existing at positions *a* and *d* to ensure a precise coiled-coil dimeric formation between α-helices from identical (homodimer) or different (heterodimer) IntFils. The core α-helix is divided further into 1A, 1B, 2A and 2B sub-domains, which play important roles in coiled-coil formation and higher-order IntFil assembly [16].

Both the homodimeric and heterodimeric coiled-coils form an antiparallel tetramer as the basic building block to form higher-order IntFil assembly units. In order to clarify further interactions between individual IntFil protomers during mature IntFil assembly, Steinert conducted crosslinking nearest-neighbor analyses of keratins—which showed four main modes of tetrameric interactions [17, 18]; these are termed A_11_ (1B–1B subdomains in phase), A_12_ (1B–2B subdomains in phase), A_22_ (2B–2B subdomains in phase), and A_CN_ (head–tail interactions) [18].

Herrmann and Aebi proposed three major assembly mechanisms of higher-order IntFil systems based on studies of lamins, vimentin, and keratins [19]. First, the assembly method of lamin was proposed to include longitudinal formation between parallel homodimers in the A_CN_ mode—which then enables multiple long strings of lamin to associate laterally through modes A_11_, A_12_, and A_22_. Second, in contrast, the vimentin method of assembly was proposed that parallel homodimers formed tetramers in antiparallel fashion—using A_11_, A_12_, A_22_ modes, followed by lateral interaction between tetramers to form the unit length filament (ULF). The ULF comprises 32-mers (i.e., eight tetramers) and is further assembled longitudinally through A_CN_ to form a mature vimentin filament. Third, in contrast to vimentin, for keratins both longitudinal and lateral filament assembly apparently happen concomitantly.

These assembly mechanisms were proposed, based on data from negative-stain electron microscopy studies which characterized the in vitro formation of keratins, lamin, and vimentin under physiological conditions [20–22]. Stemming from the “divide-and-conquer” ideology from Strelkov, extremely helpful insights into the molecular mechanisms of IntFil assembly were gained by close examination of atomic-resolution crystal structures of lamin and vimentin, and, to a lesser extent, keratins [18, 23]. Recently, the Coulombe, Bunick, and Park groups demonstrated, at the level of atomic resolution, how the A_22_ and A_11_ modes function in keratin, vimentin, and lamin assembly [16, 24, 25].

Regardless of the proposed mechanism of assembly, it is clear that IntFils form homodimeric or heterodimeric pairs, termed interaction pairs [18]. Similarly, keratin tetramers, the basic building blocks of keratin IntFils, are formed by the antiparallel interaction of two heterodimeric complexes—each comprising one type I and one type II keratin protein (e.g., KRT1/KRT10, KRT5/KRT14, KRT8/KRT18) [5, 26, 27]. One side of the keratin heterodimer has a predominantly hydrophobic character, and this forms the major interface between heterodimers in the tetrameric complex [16]; this hydrophobic interface contains a “knob-pocket tetramerization mechanism” on the type II keratin, which is key for driving the A_11_ tetrameric alignment. This interface between heterodimers is crucial for mature IntFil assembly, as demonstrated by an in vitro study of mutations in type II keratin proteins, which resulted in defective IntFil formation [16].

Given that the IntFil group is quite large, here we limit our discussion primarily to type I and type II keratins. Keratins exhibit unique and interesting evolution, expression patterns, and relevance to human disorders, which we discuss in detail (vide infra). We direct the readers to other informative reviews for a thorough discussion of types III [28], IV [29], V [30] and VI [31] IntFil families.

## Main text

### Evolutionary expansion of keratin genes

Keratins were the first group of IntFils to have their X-ray diffraction pattern discovered [1]. However, from a structural perspective, their molecular functions have been difficult to elucidate; this is in part due to the ability of keratins to form both stable heterodimers and homodimers in vitro—which led to the assumption that this can occur in the living cell (although this has been difficult to confirm) [6].

**Fig. 1:**
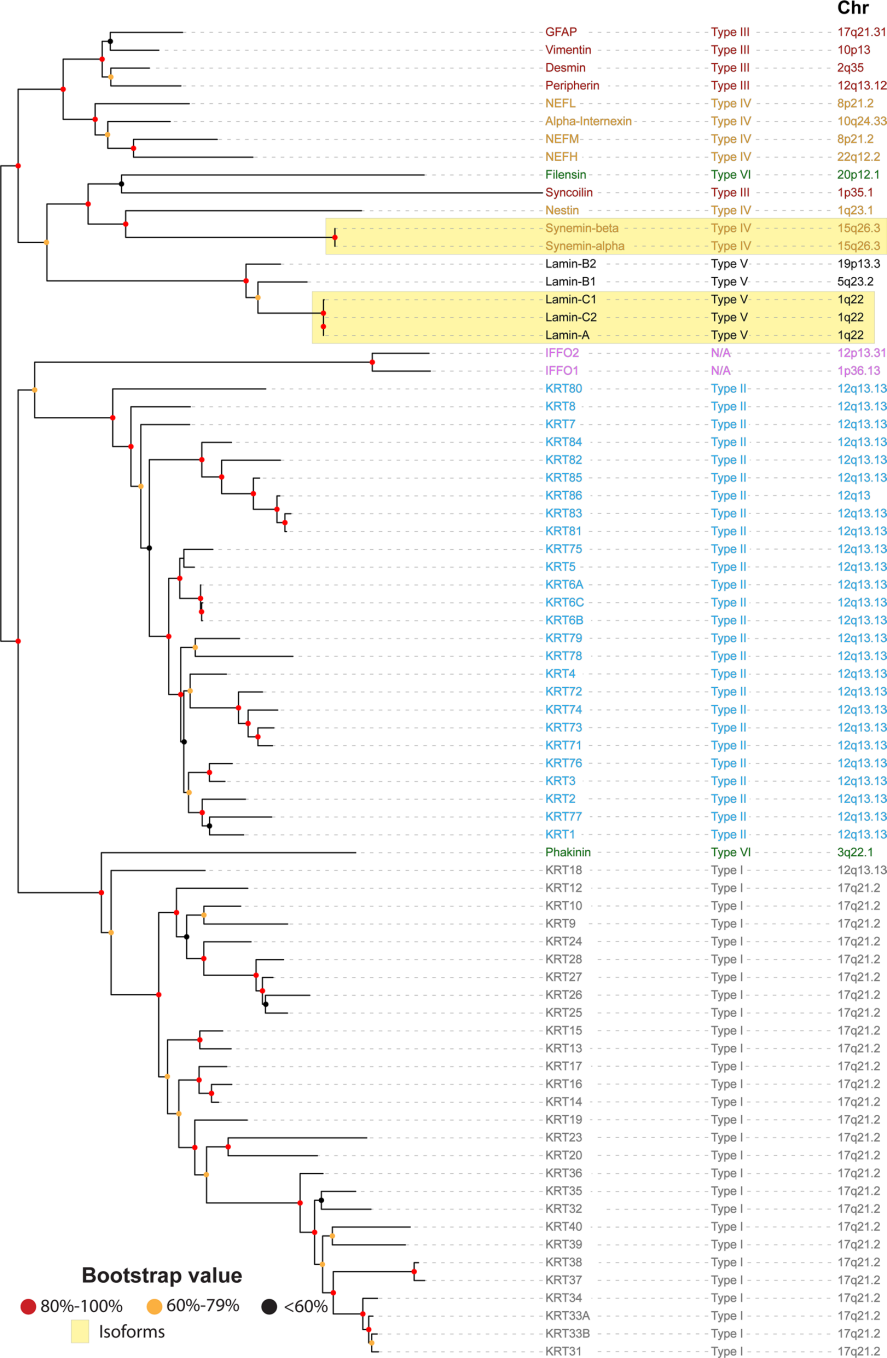
Rooted phylogenetic tree of the human (*Homo sapiens*) intermediate filaments (IntFils). Protein sequences of the 54 human IntFil types I, II, III, IV, V and VI were retrieved from the Human Intermediate Filament Database and aligned—using maximum likelihood ClustalW Phyml with bootstrap values presented at the node: > 80%, red; 60–79%, yellow; less than 60%, black. Branches of the phylogenetic tree are seen at left. The IntFil protein names are listed in the first column. Abbreviations: GFAP, glial fibrillary acidic protein; NEFL, NEFH, and NEFM correspond to neurofilaments L, H & M respectively; KRT, keratin proteins; IFFO1, IFFO2 correspond to Intermediate filament family orphans 1 & 2 respectively. The IntFil types are listed in the second column and are color-coded as follows: Type I, grey; Type II, blue; Type III, red; Type IV, gold; Type V, black; Type VI, green, and N/A, non-classified, pink. Chromosomal location of each human IntFil gene is listed in the third column. Known isoforms of synemin and lamin are denoted by the two yellow boxes

A phylogenetic tree of the human IntFil group (Fig. 1) reveals that all 18 IntFil genes of types III, IV, V and VI appear to be evolutionarily older than the keratin gene subsets (i.e., IntFil types I & II). It should be noted that the two synemin protein isoforms in the tree originate from one gene, and the three lamin isoforms are derived from one gene. Note that the IntFil genes of subgroups III, IV, V and VI are scattered among twelve chromosomes (Chr 1, 2, 3, 5, 8, 10, 12, 15, 17, 19, 20, 22); this is further evidence that these four IntFil subgroups are evolutionarily very ancient.

The human type II keratin subgroup of 26 genes (Fig. 1) is clustered entirely at Chr 12q13.13, and 27 of the 28 type I keratin genes are clustered at Chr 17q21.2 [32, 33]; the type I *KRT18* gene is an exception, located within the type II cluster at Chr 12q13.12. It remains unknown why each of these two clusters have remained together, each on a distinct chromosomal segment. Interestingly, the type I and type II clusters appear to have arisen close to the same evolutionary time. However, the phylogenetic tree suggests that the type I subset might have appeared earlier than the type II subset. This possibility is supported by additional data [vide infra].

**Fig. 2:**
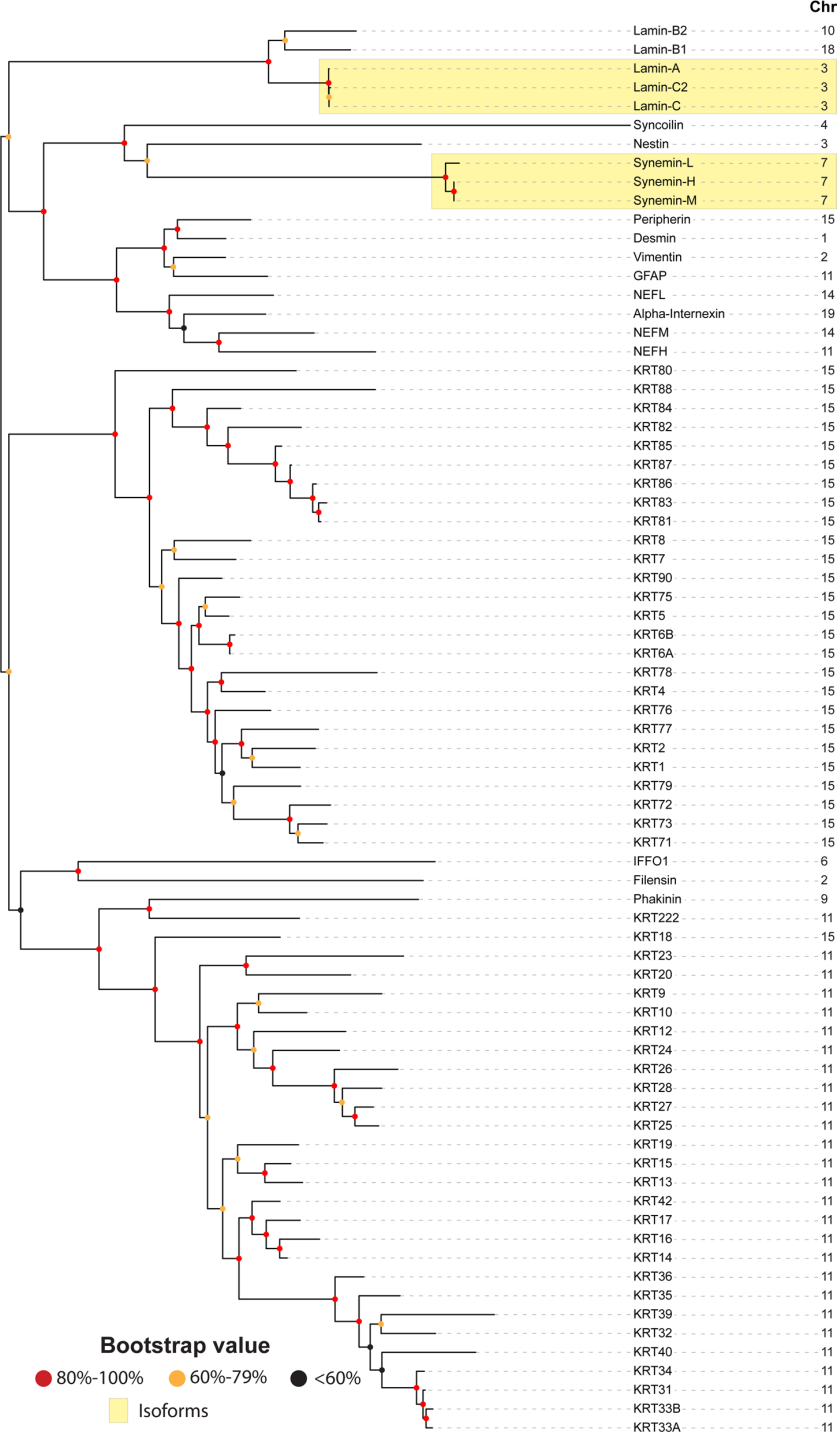
Phylogenetic tree of the inbred C57BL/6J mouse (*Mus musculus*) IntFil proteins. The same procedures were carried out here as described in the Fig. 1 legend. The IntFil protein names are listed in the first column. Abbreviations: GFAP, glial fibrillary acidic protein; NEFL, NEFH, and NEFM correspond to neurofilaments L, H & M respectively; KRT, keratin proteins; IFFO1 corresponds to IntFil family orphan 1; the evolutionarily most closely related to IFFO is filensin type VI. Chromosomal location of each mouse IntFil gene is listed in the second column. Known isoforms of lamin and synemin are denoted by the two yellow boxes

A comparable phylogenetic tree in mouse (Fig. 2) shows an evolutionary pattern that is strikingly similar to that in human—except there are 17 IntFil genes (instead of the 18 found in human) in subfamilies III, IV, V and VI that are scattered among thirteen chromosomes (Chr 1, 2, 3, 4, 6, 7, 9, 10, 11, 14, 15, 18, 19). In the mouse tree we have included three lamin protein isoforms originating from one gene and three synemin isoforms derived from one gene. The *IFFO2* IntFil gene, which is present in human, is absent in mouse; this reflects either a gene-duplication event in the human ancestor or a gene-deletion event in the mouse ancestor, after the human-mouse split ~ 70 million years ago.

The mouse *Bfsp2* gene encoding type VI phakanin, located on Chr 9, appears to be associated more closely with the type I cluster in Fig. 2, as was seen with the human phakanin gene (at 3q22.1). The other mouse type VI gene (*Bfsp1*, encoding filensin) is on Chr 2; the human filensin gene is located at Chr 20p12.1.

With regards to the keratin family, *KRT3*, *KRT37*, *KRT38,* and *KRT6C* are absent from the mouse genome. In contrast, orthologs of *KRT42*, *KRT87*, *KRT88*, *KRT90*, and *KRT222* are present in the mouse genome. The mouse type II keratin subgroup of 26 genes (Fig. 2) is located entirely on Chr 15, and 27 out of the 28 type I keratin genes are located on Chr 11. As found in human, the one exception in mouse is the type I *Krt18* gene, which is located on Chr 15 within the type II cluster; whatever caused this one particular type I gene to be located within the type II cluster in both the human and mouse genomes—while maintaining greater homology with the type I genes—must have taken place before the human-mouse split. All mouse keratin type I and type II genes are syntenic with their human orthologs [https://www.mun.ca/biology/scarr/MGA2-11-33smc.html]. Examination of keratin genes in all seven additional nonhuman mammals (chimpanzee, macaque, pig, dog, cat, cow, horse) currently registered in the Vertebrate Gene Nomenclature Committee (VGNC, vertebrate.genenames.org) reveals that the two major keratin gene clusters are also conserved in all these species.

### Duplications and diversifications of keratin genes

Paralogs are gene copies created by duplication events within the same species, resulting in new genes with the potential to evolve diverse functions. An expansion of recent paralogs that results in a cluster of similar genes—almost always within a segment of the same chromosome—has been termed ‘evolutionary bloom’. Examples of evolutionary blooms include: the mouse urinary protein (MUP) gene cluster, seen in mouse and rat but not human [34, 35]; the human secretoglobin (SCGB) [36] gene cluster; and various examples of cytochrome P450 gene (CYP) clusters in vertebrates [37] and invertebrates [37, 38].

**Fig. 3:**
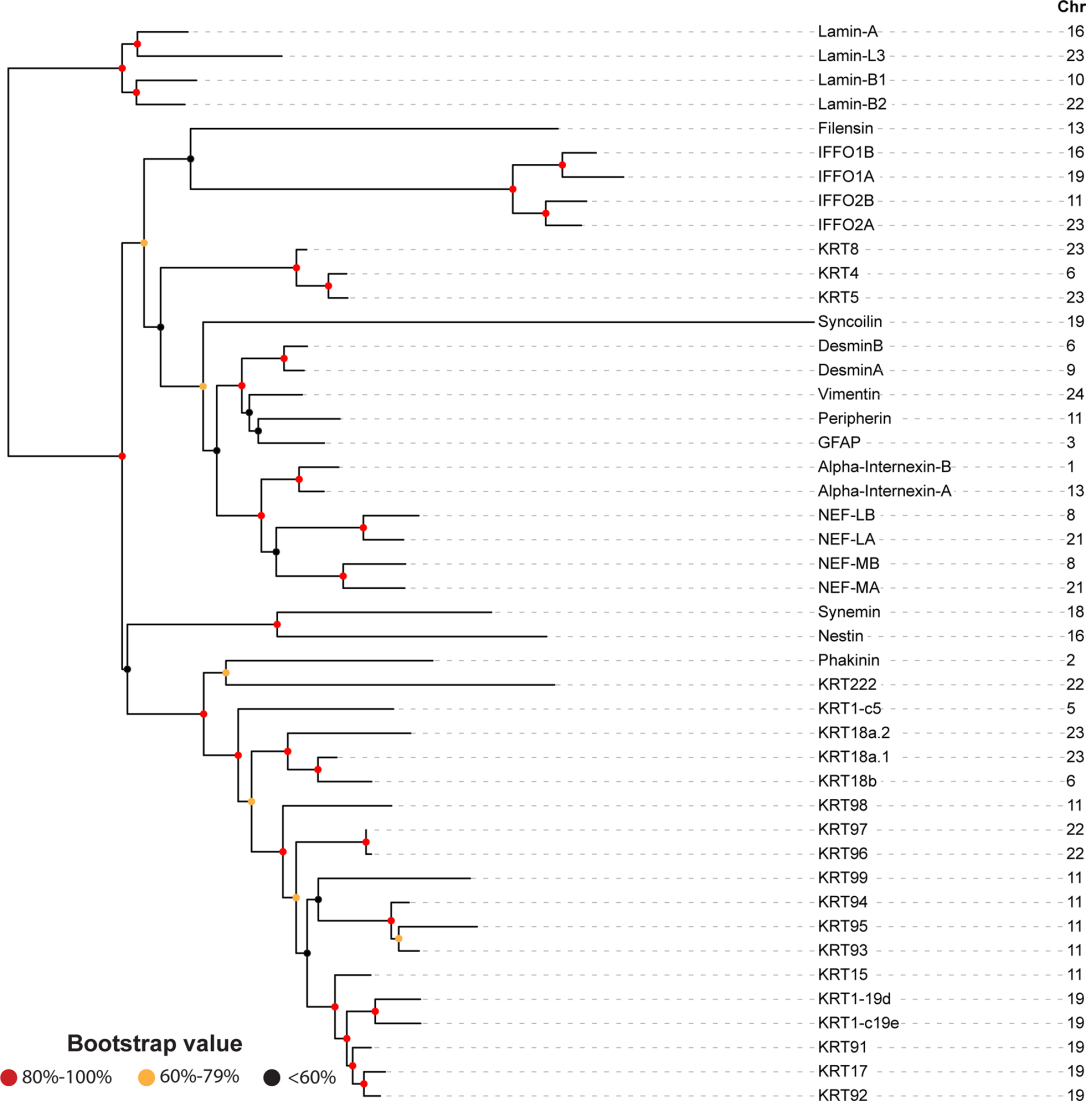
Phylogenetic tree of the zebrafish (*Danio rerio*) IntFil proteins. The same procedures were carried out here as described in the Fig. 1 legend. The IntFil protein names are listed in the first column. Abbreviations: GFAP, glial fibrillary acidic protein; NEF-LA, NEF-LB, NEF-MA and NEF-MB correspond to neurofilaments LA, LB, MA & MB respectively; KRT, keratin proteins; IFFO1A, IFFO1B, IFFO2A & IFFO2B correspond to four IntFil family orphans. Chromosomal location of each IntFil gene is listed in the second column. KRT1-c5, KRT1-19d, and KRT1-c19e are keratin type I gene c5, 19d, and c19e respectively (they are not keratin 1)

Are these keratin gene evolutionary blooms seen in the fish genome? Fig. 3 shows a comparable phylogenetic tree for zebrafish. Compared with human IntFil genes (18 non-keratin genes and 54 keratin genes) and mouse IntFil genes (17 non-keratin genes and 54 keratin genes), the zebrafish genome appears to contain 24 non-keratin genes and only 21 keratin genes (seventeen type I, three type II, and one uncharacterized type). Interestingly, the type VI *bfsp2* gene (encoding phakinin), which functions in transparency of the lens of the zebrafish eye [39], is more closely associated evolutionarily with keratin genes than with the non-keratin genes; this is also found in human and mouse—which diverged from bony fish ~ 420 million years ago. The other type VI IntFil gene in mammals, *BFSP1* (encoding filensin) that is also involved in lens transparency [39], appears not to have an ortholog in zebrafish.

**Fig. 4:**
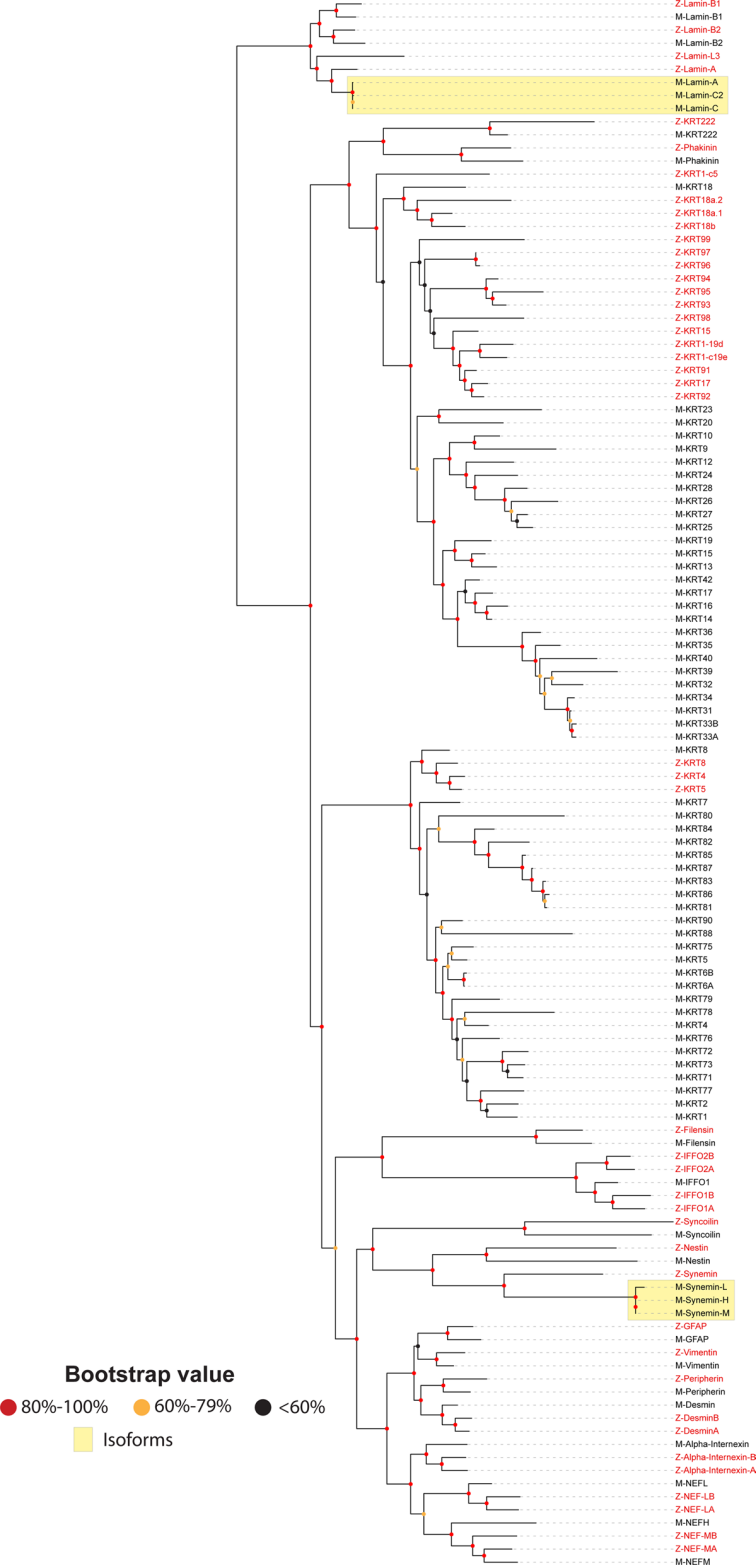
Phylogenetic tree of the zebrafish IntFil proteins superimposed on the mouse phylogenetic tree. Names of zebrafish proteins are in red font, mouse proteins in black font. The same procedures were carried out here, as described in the Fig. 1 legend. The IntFil protein names are listed in the first column. “M-” or “Z-” precedes mouse and zebrafish IntFils, respectively. Abbreviations are the same as Figs. 2 and 3. Known isoforms of mouse lamins and synemins are denoted by yellow boxes. The zebrafish’s KRT1-c5, KRT1-19d, and KRT1-c19e are keratin type I gene c5, 19d, and c19e respectively (they are not keratin 1)

Although five keratin genes appear on zebrafish Chr 19, and six keratin genes appear on Chr 11, there is no definitive evidence of an evolutionary bloom here (Fig. 3). If one superimposes zebrafish IntFil proteins on the mouse IntFil proteins in the same phylogenetic tree (Fig. 4), the 24 zebrafish non-keratin proteins show highest homology with the 17 mouse non-keratin proteins; and the 18 zebrafish type I keratin proteins reveal highest homology with the 26 type I keratin proteins in mouse, whereas the three zebrafish type II keratins show highest homology with mouse type II KRT8. These data suggest that both acidic type I and basic type II keratins appeared before the land-sea animal divergence ~ 420 million year ago, and both the type I KRT18 and type II KRT8 resemble most closely the ancestral precursor of all other keratins [40].

Furthermore, the basic type II keratin genes might have experienced more selective pressure causing massive gene loss in bony fish, in agreement with a previous report [41], because the type II keratin group in zebrafish has far fewer genes compared with the type I group. Figures 1, 2 and 3 thus suggest that numerous independent gene-duplication events—specifically in the case of the type II keratin cluster of human and mouse keratin genes—occurred evolutionarily before the human-mouse split but after the sea-to-land animal transition.

A gene-duplication event resulting in paralogs is, in and of itself, a selected characteristic, with rates of gene duplication varying across the Tree of Life. Despite being potentially disruptive at both genome and expression levels, the ability of genes to duplicate likely persists as an evolutionarily beneficial device, because it provides species with flexible mechanisms of introducing genetic heterogeneity and allowing members to adapt and thrive during the myriad shifts in environmental pressures experienced by land animals.

From the viewpoint of gene regulation along the linear chromosome, why might evolutionary blooms appear and persist during evolution? One reason for an urgent requirement for many new keratin paralogs—is most likely the critical need for new species of land animals to survive and thrive in the midst of new environmental pressures. There is a second reason. Over a few millions of years, *cis*-regulatory sequences in noncoding regions (i.e., introns, promoters, enhancers, usually within 10 to 200 kb of the original regulated gene) might control expression of some, or many, parologous genes located nearby on the same chromosomal segment [42, 43]. In contrast, single gene-duplication events, taking place over much longer periods of evolutionary time, more likely have established their own distinct *cis*-regulatory noncoding regions—thereby not needing to remain as a cluster at one chromosomal segment; examples would include the type III, IV, V and VI IntFil genes.

### Evolution of keratin gene functions

**Fig. 5:**
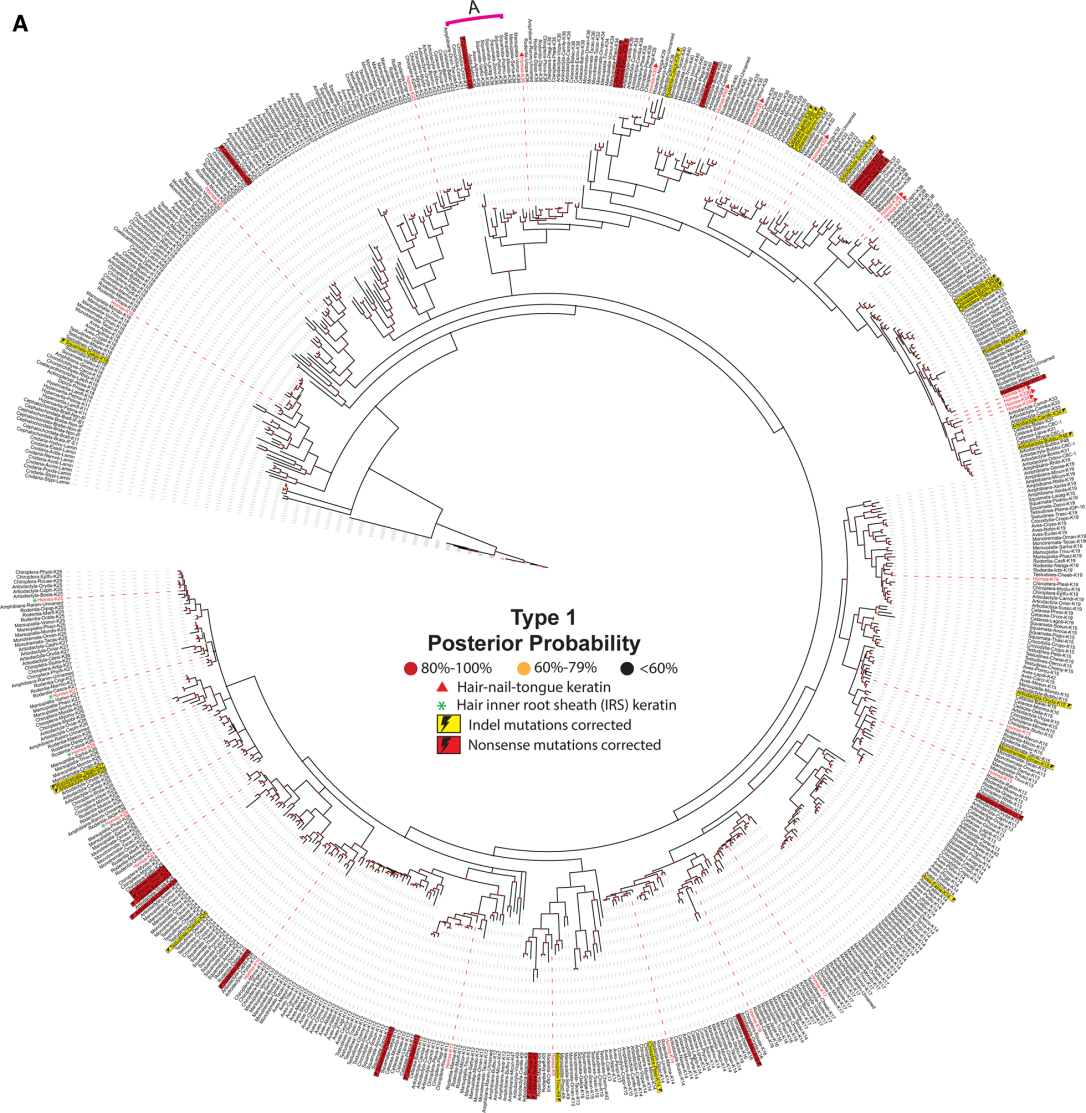

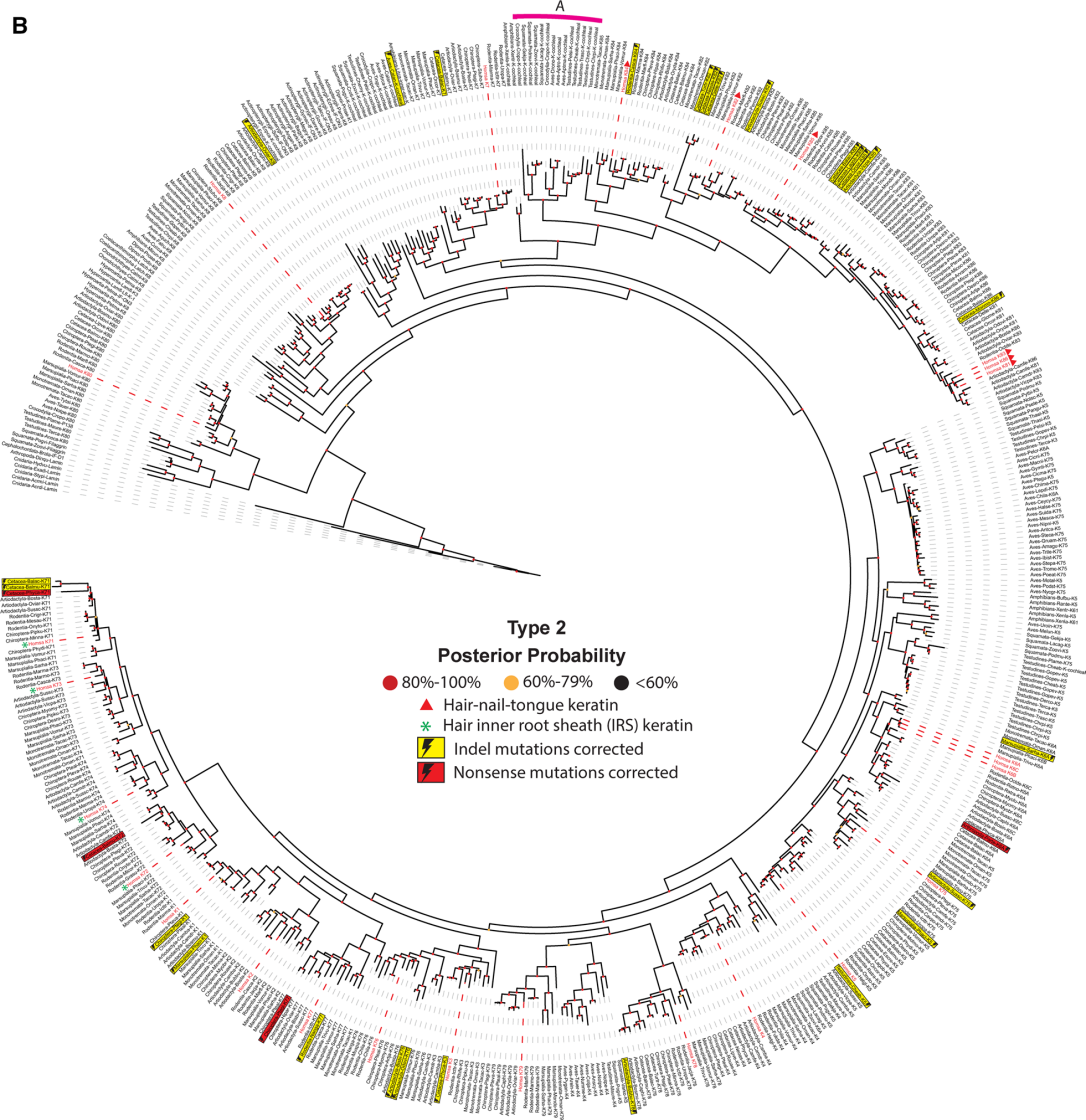
Evolution of animal keratins. Evolutionary relatedness in the type I (**a**) and II (**b**) keratin protein sequences from a broad representation of animal species, including human, was reconstructed. The 20 Phyla (or Classes or Orders) that were chosen include: *Actinopterygii*, ray-finned fishes; *Amphibian*, frogs-toads-salamanders; *Arthropoda*, insects-arachnids-millipedes-crusteaceans; *Artiodactyla*, ungulates (hoofed animals); *Aves*, birds; *Cephalochordata*, anphioxus; *Cetacea*, marine mammals; *Chiroptera*, bats & flying foxes; *Chondrichthyes*, cartilagenous fishes; *Cnidaria*, jellyfish; *Coelacanthimorpha,*, lobe-finned fishes with rudimenary legs; *Crocodylia*, crocodiles-alligators; *Dipnoi*, lungfish; *Homo sapiens*, modern-day humans; *Hyperoartia*, lampreys-eels; *Marsupialia*, kangaroos-wallaby-koalas-oppossums-wombats; *Monotremata*, platypus-echidna; *Rodentia*, mice-rats; *Squamata*, lizards-snakes; and *Testudines*, turtles, tortoise, terrapins. Protein sequences included in the reconstruction were identified by using the basic local alignment search tool (BLAST) on human keratin proteins against each non-redundant protein database for the clades of interest. For clades more distantly related evolutionarily to humans than *Amphibia*, only the protein with the highest similarity to human, as determined by the BLOSUM 62 matrix, was included. For *Amphibia* and clades more closely related to humans than amphibians, the top three proteins with the highest similarity to human—as determined by the BLOSUM 62 matrix—were used for analysis. Evolutionary relationships were inferred using MrBayes under a mixed amino acid model and visualized with the Interactive Tree-of-Life [accessed at itol.embl.de]. The dashed lines link the keratin proteins with their corresponding label. Human keratins are indicated by a red dashed line and red font. Known isoforms are denoted by the yellow boxes. *Cnidaria* was used as the root for both phylogenetic trees. Labels are written as follows: clades, species, protein name. The “PREDICTED: LOW QUALITY” proteins were labeled with their corrected mutations: yellow lightning bolt indicates insertion/deletion (indel), red lightning bolt indicates nonsense mutation. Clade A is indicated by a pink line. Nodes are colored to indicate posterior probabilities: red, 80–100%; yellow, 60–79%; black, < 60%. Details on the animal proteins represented in this phylogenetic tree are contained in Additional file 1: Table S1 and Additional file 2: Table S2 (for type I and type II respectively)

Screening 259 species and subspecies in 20 phyla of animals, from jellyfish to human, we examined various features found in type I (Fig. 5a) and type II (Fig. 5b) keratin proteins; we also studied when during the evolutionary history of keratins these features have apparently arisen, disappeared, and, on occasion, reappeared. Between ~ 380 and ~ 150 million years (from lungfish to monotremes), dozens of new forms of type I and type II keratin proteins were rapidly co-opted to participate in successfully creating new anatomical structures that were needed in the transition of sea animals to land animals.

The mammalian keratin group members have highly similar rod domains—that are uniquely expressed throughout the epidermis, epithelial cells, and hair follicle. This suggests that small differences among keratin primary sequence are highly specific to a tissue type; this hypothesis is supported by crystallographic data showing that unique amino acids belonging to keratin interaction pairs are primarily positioned along the outer edges of the coiled-coil rod domain, in order to maximize diversity of surface chemistry of the IntFil filament [44].

However, specialized expression, or pairing of IntFil proteins, is not always straightforward. For example, *Cetaceans* (e.g., whale, dolphin, porpoise) lack expression of KRT24, but, in its absence, the putative partners of KRT24 (i.e., KRT3 and KRT5) interact with KRT14 and KRT12. This finding indicates that keratin proteins can become dispensable in some species, while being repurposed in others [45, 46].

We created phylogenetic trees for type I and type II keratin proteins from a broad representation of animal species (Fig. 5). These data suggest that the clade containing the KRT18 (type I) and the KRT80 and KRT8 (type II) proteins is least divergent from the ancient IntFil protein lamin, and most closely resembles precursors for the other members of the keratin group. Localization of the majority of IntFil proteins from earlier Phyla, Classes or Orders (i.e., *Cnidaria*, *Arthropoda*, *Cephalochordata*, *Hyperoartia*, *Chrondrichthyes*, *Actinopterygii*, *Coelacanthimorpha* and *Dipnoi*) closest to the ancient protein lamin, and closest to the KRT18, KRT80, and KRT8 clade strengthens the hypothesis that these three keratins were likely the first keratins to form the embryonic epithelium in the *Animalia* Kingdom [47, 48].

The Fig. 5 trees also suggest that the keratins in species diverging early—relative to human (i.e., *Cnidaria* and *Arthropoda*)—have a higher number of proteins related to the ancient IntFil protein, lamin, than to keratins. Within our data, *Arthropoda* appears to have only one type II keratin (KRT6A) and two type I keratins (KRT13 and KRT14). The type II KRT80 protein in *Cnidaria* (jellyfish) is apparently lost and then does not reappear until the *Testudines* Order (turtle). These findings are consistent with the notion that keratin genes can be lost, gained and/or repurposed [45, 46].

The type I and type II keratins encoded in the amphioxus (*Cephalochordata*) genome are also mostly comprised of lamin-like proteins. In contrast, the type I and type II keratins in lamprey (*Hyperoartia*), cartilaginous fish (*Chondrichthyes*), and lobe-finned fish with rudimentary legs (*Coelacanthimorpha*) are closely related to ancestors of type I KRT18 and type II KRT8. Ancestors of the KRT18 and KRT23 type I proteins most likely led to the type I keratins in ray-finned fish (*Actinopterygii*) and lungfish (*Dipnoi*). Ray-finned lish and lungfish type II keratins are less divergent from ancestors of the KRT8 proteins.

In the *Amphibia* Class, type I keratins are closely related to ancestors of 14 keratins (KRT12, KRT17, KRT18, KRT19, KRT20, KRT23, KRT25, KRT26, KRT27, KRT28, KRT32, KRT36, KRT39, KRT40), whereas type II keratins are closely related to ancestors of KRT8, KRT7, KRT6A, 6B, and 6C. The type I keratins in *Amphibia* are strikingly diverse; these observations are consistent with an early split of the phylogenetic tree concordant with the species tree, followed by multiple duplications with subsequent variation and selection. Given that this observation is not replicated in *Amphibia* type II sequences, it could be posited that type II keratins have broadly experienced more selective pressure, while type I keratins are more robust in structural variation.

The phylogenetic trees also suggest that the earliest hair-nails-tongue (KRT32, KRT36, KRT39, KRT40) and hair inner-root-sheath (IRS) keratins (KRT25, KRT26, KRT27, KRT28) appear to have evolved from the type I keratin in *Amphibia* ancestors (Fig. 5a). The data presented in these phylogenetic trees thus support the previous suggestions that the hair-nails-tongue keratins first appeared in tetrapods (i.e., all vertebrates evolutionarily later than fishes) [49]—to provide protection from friction caused by terrestrial movement and/or to prevent dehydration [49, 50]. Furthermore, the Fig. 5 trees show that major members of the hair-nails-tongue keratin group (type I: KRT31, KRT32, KRT33A, KRT33B, KRT34, KRT35, KRT36, KRT37, KRT38, KRT39, KRT40; type II: KRT81, KRT82, KRT83, KRT84, KRT85, KRT86) are less divergent from the KRT18, KRT80, and KRT8 ancestral precursors than the group of hair-IRS keratin (type I: KRT25, KRT26, KRT27, KRT28; type II: KRT71, KRT72, KRT73, KRT74); these findings suggest that the hair-nails-tongue, and the hair-IRS, groups appear to have co-evolved, first appearing in the Order *Amphibia* (Fig. 5a, b). Collectively, these phylogenetic trees support the hypothesis that the massive appearance of ecological function of keratins started in *Amphibia*, which corresponds to the transition from a water to land lifestyle [50].

Intriguingly, the Fig. 5 data also indicate that the *Amphibia* ancestral hair-IRS type I keratins (KRT25, KRT26, KRT27, KRT28) and hair-nails-tongue type I keratins (KRT32, KRT36, KRT39, KRT40) disappeared in the *Sauropsida* clade (*Testudines, Crocodylia, Aves*, and *Squamata*) and reappeared again in the Class *Mammalia*. There are a small number of proteins—from *Crocodylia, Aves*, *Testudines* and *Squamata*—that appear to share the same common ancestor with the mammalian hair-nails-tongue keratins, though they are not directly related (Fig. 5a, b, *Clade A*). It is likely that this reflects the huge molecular difference between the *Sauropsida* β-keratin and the mammalian α-keratin and β-keratin; this also reflects the large differences in skin appendages between *Sauropsida* (feather, scale, beak and claw) and *Mammalia* (hair, scale, claw, horn, hoof, and nail) [50].

With regard to marine mammals (i.e., *Cetaceans*)—the suprabasal plantar-specific keratin genes (type I: *KRT10*; type II: *KRT1*, *KRT2*, *KRT77*) and sweat gland-specific keratin gene (type I *KRT9*) are absent or truncated, whereas only basal keratin genes (type I *KRT14*; type II *KRT5*,) and hyperproliferation-signal-specific keratin genes (type I *KRT17*; type II *KRT6A,B,C*,) are found in the *Cetacean* genome [51]. This discovery is correlated with the fact that aquatic mammals have thicker basal keratinocyte layers than terrestrial mammals, and that *Cetaceans* lack the need for footpads and sweat glands (Fig. 5). Note again, that although some keratins are conserved, others have disappeared, reappeared and/or apparently new ones have arisen—due to the natural selection pressures that facilitate adaptation of new cell type-, tissue- and organ-specific formation; this phenomenon is fundamental in evolution.

Another fascinating example of a missing keratin protein is the absence of the type I keratin KRT24 in whale and walrus—a feature that is thought to play a role in the evolutionary adaptation of these species. Comparative genomics studies have suggested that *KRT24* originated in a common ancestor of *Amniotes* (a clade of tetrapod vertebrates), but then was lost independently in three clades of mammals (i.e., camels, cetaceans, and a subclade of pinnipeds including the eared-seal and walrus) [45, 46]. At first glance, our data (Fig. 5a) would seem to contradict these reports; however, a closer inspection of the *Cetacean KRT24* gene sequence revealed that it contains multiple premature stop codons. These would likely result in either elimination of the messenger RNA by nonsense-mediated decay, or production of a non-functional protein that would rapidly undergo proteasomal degradation. The existence of these premature stop codons in the sequence of *KRT24* in *Cetaceans* supports the notion that *KRT24* is dispensable; this discovery also may provide a mechanism by which keratins ‘disappear’ from the genome (i.e., slow accumulation of mutations) [52]. Furthermore, from our phylogenetic tree, we have found the possible existence of truncated KRT32, KRT39 and KRT40 proteins in the *Cetacean* group; these findings suggest further the mutational inactivation of these keratins among the members of the Infraorder *Cetacea*.

In conclusion, the appearance-disappearance-reappearance of keratin features—throughout evolutionary history—support the notion that the gain-of-function and loss-of-function of certain types of keratins (Fig. 5) are likely to be involved in evolutionary adaptation [45]. If the same rigorous examination across the *Animalia* Kingdom—as was done here for the keratin clusters (Fig. 5)—were to be carried out for the MUP [34, 35], SCGB [36], and CYP [37, 38] evolutionary blooms, perhaps similar patterns of gain-of-function and loss-of-function (as a function of evolutionary time) might also become apparent. Consistent with the observations of a higher tendency of truncated keratins appearing in the type I keratins, the rates of evolution of new keratin proteins, specifically type I, coincide with the rates of evolution of all metazoans, and, ultimately, mammals.

### Tissue-specific expression of human keratins

#### Tissue-specific expression patterns of keratin pairs

**Fig. 6:**
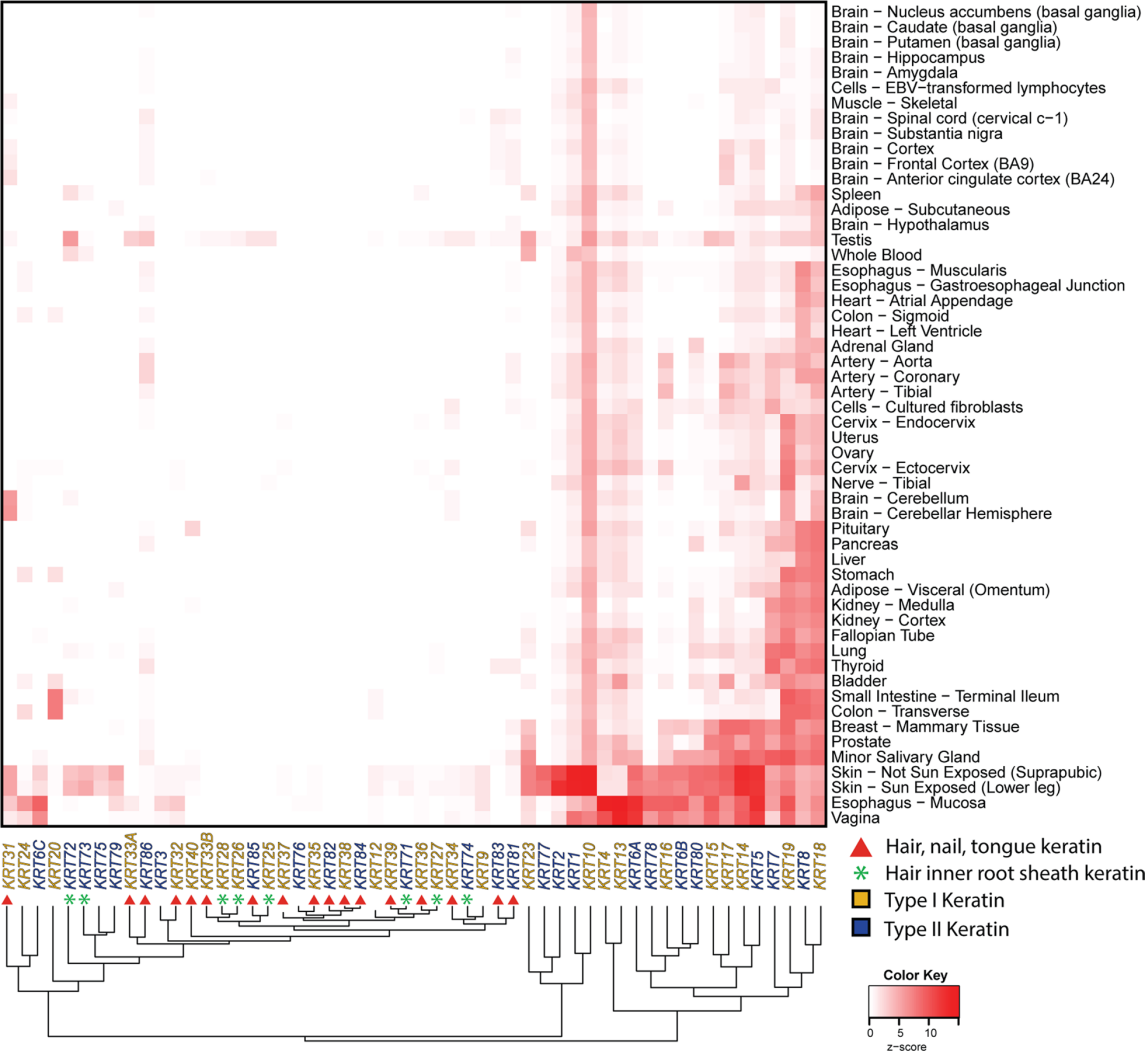
Tissue-specific keratin expression in adult human tissues. Median transcripts per million (TPM) expression values for keratin genes in 54 human tissues were retrieved from the GTEx database [53] and displayed as a heatmap—with keratin proteins listed across the bottom and human tissues on the *Y*-axis at right. The phylogenetic clustering of keratin gene expression is displayed along the *X*-axis at bottom. Data are logarithm base-10 (value + 1) transformed, scaled by row, and presented as a *z*-score with white tiles representing low or no expression and red tiles representing high expression. Keratin genes (columns) and human tissues (rows) were clustered using the maximum distance and complete clustering methods. Keratin genes are color-coded to indicate type I (gold) or type II (blue) keratin. Hair-nails-tongue keratin genes are denoted by a red circle. Hair-inner-root-sheath keratin genes are indicated by a green star

Using data retrieved from the Genotype-Tissue Expression (GTEx) project [53], we reconstructed the expression of keratins throughout the human body in a tissue-specific manner (Fig. 6). Interestingly, the majority of keratin genes (i.e., *KRT3, KRT6C, KRT9, KRT12, KRT20, KRT24, KRT25, KRT26, KRT27, KRT28, KRT31, KRT32, KRT33A, KRT33B, KRT34, KRT35, KRT36, KRT37, KRT38, KRT39, KRT40, KRT71, KRT72, KRT73, KRT74, KRT75, KRT76, KRT79, KRT81, KRT82, KRT83, KRT84, KRT85, KRT86*)—lack highly substantive expression in the majority of human tissues listed in GTEx.

It is important to note that the GTEx database does not contain keratin expression data on hair, nails and tongue, which are known to be tissues with exceptionally high expression of many keratins. In fact, all keratin genes that lack marked expression in any human tissue in GTEx are those with notable expression in either hair, nails, or tongue (Fig. 6). It is likely that, if GTEx had data on these other tissues, one would see high expression for these tissues.

As anticipated, clustering of gene expression patterns revealed similarities in the tissue-specific expression patterns of the five keratin-interaction pairs (*i.e., KRT1/KRT10, KRT8/KRT18, KRT5/KRT14, KRT6/KRT16 and KRT6/KRT17* genes). However, tissue-specific expression patterns of *KRT6A, KRT6B* and *KRT6C* were only moderately similar to that of *KRT17* (vide infra). Given the importance of keratin-interaction pairs for their function, below we provide detailed discussions solely of the expression patterns for those genes involved in these five keratin pairs.

#### KRT1/KRT10

Both *KRT1* and *KRT10* display expansive expression patterns with expression in every tissue within the GTEx database (Fig. 6). This diverse expression pattern is likely due to their roles in differentiated epithelial cells [54]. However, despite their functions as a pair, the tissue-specific expression levels of *KRT1* and *KRT10* are only weakly positively correlated (*ρ* = 0.54, *P* = 2.70e-05). Even with their weak correlation, tissue-specific expression patterns between *KRT1* and *KRT10* did cluster next to one another—indicating that their expression patterns were more similar to each other than to any other keratin.

*KRT1* expression is lower than *KRT10* expression in every tissue, except for whole blood [transcripts-per-million (TPM) of 16.1 vs 10.5]. As shown in Fig. 6, *KRT10* is the most highly expressed keratin gene in subcutaneous adipose tissue, arteries (aorta and tibial), all brain regions except for cerebellum and cerebellar hemispheres, cell cultures [cultured fibroblasts and Epstein-Barr virus (EBV)-transformed lymphocytes], sigmoid colon, atrial appendage and left ventricle of heart, skeletal muscle, and skin (both sun-exposed of lower leg and non-sun-exposed of suprapubic region).

The observation of *KRT10* expression in every tissue within the GTEx database is in agreement with numerous prior reports of expression in skin [55], breast [56], testis [57], cervix [58], thymus [59] and vagina [60]; and with the finding that expression of a transgene driven by the *KRT10* promoter was observed in stomach, small intestine, cecum, colon, spleen, and pancreas [61]. While *KRT1* expression is well established in skin integrity [55, 62], colonic mucosa [63], kidney [64] and vagina [65], the GTEx data indicate that *KRT1* has a much more expansive expression pattern than is suggested by the literature. These expression data also raise the question as to whether *KRT10* is expressed in terminally-differentiated epithelial cells [66].

#### KRT8/KRT18

Both *KRT8* and *KRT18* are expressed in every tissue within the GTEx database (Fig. 6). This diverse expression pattern is likely due to their role in simple epithelial cells [54, 67]. In contrast to *KRT1/KRT10*, *KRT8* and *KRT18* tissue-specific expression levels were very strongly positively correlated (*ρ* = 0.89, *P* = 5.5e–19), and clustered next to each other. *KRT8* was the most highly expressed keratin in esophagus, both in the gastroesophageal junction and the muscularis. *KRT8* expression is greater than any other keratin in three specific locations: the gastroesophageal junction of esophagus, atrial appendage of heart, and left ventricle of heart.

Similarly, *KRT18* was the most highly expressed keratin gene in several tissues: adipose tissue (visceral omentum), adrenal gland, coronary artery, renal cortex and medulla, liver, pancreas, pituitary, spleen, and thyroid. Thus, as expected, *KRT18* expression is higher than *KRT8* in every tissue except for the aorta, bladder, esophagus (gastroesophageal junction), atrial appendage of the heart, transverse colon, and terminal ileum of small intestine.

*KRT8* expression in the GTEx database is in agreement with previous reports that described expression in uterus, vagina, bladder [60], pancreas, liver [68], fetal heart tissues [69], mammary tissue [70], colon, small intestine, esophagus, kidney, lung [71], ovary [72], stomach, thyroid and, prostate [73]. *KRT18* expression patterns in GTEx are in agreement with previous reports in bladder [54], mammary tissue [70], intestine [54, 74], pancreas [74], liver [54, 74, 75], lung [67, 75], esophagus [76], colon [54, 75, 77], kidney, cervix, spleen, brain and skin [75].

#### KRT5/KRT14

Both *KRT5* and *KRT14* are expressed in most tissues within the GTEx database (Fig. 6). Again, this is consistent with their known expression in stratified and simple epithelium [74]. Tissue-specific expression levels of *KRT5* and *KRT14* are strongly positively correlated (*ρ* = 0.81, *P* = 2.2e−13) and clustered next to one another. Similarities in their tissue-specific expression levels and patterns are expected, given their role as interaction partners in heterodimeric pairs. Neither of these keratin genes is the most highly expressed keratin in any of the tissues listed within the GTEx database. *KRT5* expression is higher than *KRT14* expression in most tissues—except for subcutaneous adipose, aorta, coronary and tibial arteries, the caudate region of brain, the spinal cord (cervical C-1), breast/mammary, minor salivary gland, skeletal muscle, tibial nerve, terminal ileum of small intestine, skin (sun-exposed and non-sun-exposed), and cultured fibroblast cells.

The *KRT5* expression pattern described in the GTEx database is in agreement with previous reports of *KRT*5 expression in differentiating adipose-derived stem cells [78], whole blood [79], esophagus, skin, bladder, mammary tissue [54, 80], cervix [81], lung [80, 82], prostate, liver, pancreas, stomach and salivary gland [80, 83]. The finding that *KRT14* expression occurs in every tissue, except for the renal medulla, is in agreement with previous reports demonstrating *KRT14* expression in uterus, vagina, bladder [60] esophagus [54], mammary tissue, lung, prostate and salivary gland [54, 80]. Furthermore, failure to find *KRT14* expression in renal medulla is consistent with a previous report [80].

#### KRT6/KRT16

As expected, tissue-specific expression levels were strongly correlated with the keratin-interaction pairings *KRT6* (*KRT6A*, *KRT6B* and *KRT6C*) and *KRT16* (Fig. 6): *KRT6A*/*KRT16* (*ρ* = 0.83, *P* = 1.1e−14); *KRT6B*/*KRT16* (*ρ* = 0.83, *P* = 1.5e−14); and *KRT6C*/*KRT16* (*ρ* = 0.80, *P* = 3.6e−13). It should be noted, however, that the correlation between *KRT6B* and *KRT16* is heavily influenced by the large number of genes having low or no expression, which will be similarly classified near the bottom of the ranked-order list.

GTEx data indicate that *KRT6A* is expressed in every tissue. In contrast, *KRT6B* is not expressed in the brain region except in cerebellum, nor is it in EBV-transformed lymphocytes, the left ventricle of heart, renal cortex and medulla, skeletal muscle, or whole blood. In addition to the same tissues that lack *KRT6B* expression, *KRT6C* is not expressed in subcutaneous or visceral (omentum) adipose, adrenal gland, cultured fibroblasts, endocervix, sigmoid and transverse colon, gastroesophageal junction of the esophagus, atrial appendage and left ventricle of heart, or the liver, lung, tibial nerve, pancreas, stomach, and thyroid.

*KRT16* is expressed in every tissue except for renal medulla, and the following brain regions: hypothalamus, frontal cortex, anterior cingulate cortex, hippocampus, caudate, nucleus accumbens, putamen, substantia nigra, and amygdala (Fig. 6). Interestingly, there were only eight tissues with higher expression of *KRT16* than any of the three KRT6 keratins: the adipose subcutaneous, aorta, coronary and tibial regions of the artery, breast mammary tissue, cervix endocervix, tibial nerve, and prostate (Fig. 6).

The finding that KRT6 genes (*KRT6A*, *KRT6B* or *KRT6C*) are expressed in every tissue is in agreement with previous reports of KRT6 expression in uterus, vagina, bladder [60, 80], skin [54, 84], esophagus, liver, lung, pancreas, prostate, salivary gland, and stomach [54, 80]. That *KRT16* expression is found in most tissues is consistent with previous reports of expression in cervix [85], esophagus [54], and skin [86]. However, the expansive *KRT16* expression pattern in human tissues in GTEx is in disagreement with previous reports that failed to find *KRT16* expression in hepatocytes, colon, small intestine, mammary gland ducts [54], bladder [54, 87], and prostate [88]. Interestingly, the expression pattern of *KRT16* is shown to be more closely related to that of *KRT6A* and *KRT6B* than to the expression pattern of *KRT17*.

#### KRT6/KRT17

Given that KRT6 and KRT17 are an interaction pair, it was unexpected to see *KRT17* expressed in every tissue, whereas only *KRT6A* (and not *KRT6B* or *KRT6C*) is similarly expressed in every tissue (Fig. 6); however, their tissue-specific expression levels were only weakly positively correlated (*ρ* = 0.59, *P* = 2.6e−6). Despite the high number of tissues having undetectable *KRT6B* and *KRT6C* expression, both genes exhibited weakly positive correlations in tissue-specific expression patterns with *KRT17* (*KRT6B* = *ρ* = 0.61, *P* = 6.8e−7; *KRT6C* = *ρ* = 058, *P* = 5.1e−6). Interestingly, the strengths of these correlations are almost identical to those of *KRT6A* and *KRT17*; this is likely due to the fact that tissues having low or no expression will similarly be ranked consistently near the bottom. This would result in correspondingly weak positive correlations.

However, when comparing tissue-specific expression patterns between *KRT17* and *KRT6A*, *KRT6B* or *KRT6C* by analyzing their clustering patterns, it became apparent that *KRT17* and *KRT6A* are more similar than *KRT17* and either *KRT6B* or *KRT6C*. *KRT17* expression is higher than *KRT6B* or *KRT6C* expression in every tissue within the GTEx database, except for the muscosal esophagus and vagina. *KRT17* expression is higher than *KRT6A* expression in every tissue in the GTEx database—except for subcutaneous and visceral (omentum) adipose, the cerebellum and nucleus accumbens (basal ganglia) of brain, ectocervix, transverse colon, gastroesophageal junction, mucosa and muscularis of the esophagus, Fallopian tube, atrial appendage and left ventricle of heart, liver, skeletal muscle, ovary, pancreas, terminal ileum of the small intestine, spleen, stomach, uterus, and vagina.

The discovery that *KRT17* is expressed in every tissue in GTEx is in agreement with previous reports of *KRT17* expression in skin, esophagus, mammary gland [54], bladder, prostate [89], lung [90], and ovary [91]. However, the expansive *KRT17* expression that we found in the GTEx database is different from previous reports that failed to detect *KRT17* expression in colon, small intestine, liver, salivary gland, esophagus, stomach, intestine [54, 89], cervix [92], and thyroid [93].

#### Possible reasons for discrepancies

The data that we have collected from GTEx disagree with some of the findings from previous publications. The main reason is undoubtedly due to advances in imaging and scientific methodology. Indeed, most of the previous findings were derived from immunostaining to detect the signal of protein expression only and failed to detect**:** KRT10 in bladder [94] or uterus epithelial cells [60]; KRT8 in cervix, spleen, or testis [58, 73]; KRT16 in colon, small intestine, mammary gland duct [54], bladder [54, 87], or prostate [88]; KRT17 in colon, small intestine, liver, salivary gland, esophagus, stomach, intestine [54, 89], cervix [92], or thyroid [93].

Moreover, some older studies used 2D-electrophoresis, which is much less sensitive and can give false-negative signals; for example, neither KRT5 nor KRT14 expression was detected in brain, muscle, ovary, pancreas, spleen, or testis [80]. Furthermore, lack of knowledge about highly similar proteins meant gene-specific probes were not used; for example, in the case of KRT6, this could limit the ability to correctly distinguish the expression of *KRT6A*, *KRT6B*, and *KRT6C* [95].

It is also possible that a false-positive signal might arise from the use of anti-keratin antibodies that have similar epitopes, e.g., using anti-KRT5/KRT6 to detect KRT5 or KRT6 [95]. Recently, Marc’s group identified possible cross-contamination occurring for the GTEx data during library preparation [96]. This certainly raises concerns regarding validity of some data presented in the GTEx database, and it will be important—as newer large-scale datasets emerge—to cross-validate the keratin gene expression findings described herein from GTEx.

### Involvement of keratin proteins in human disease

In the ClinVar database, analysis of human disease-causing variations within keratin proteins reveals that for diseases of the skin—such as epidermolytic ichthyosis, superficial epidermolytic ichthyosis, epidermolysis bullosa simplex, palmoplantar keratoderma, and white-sponge nevus—more changes occur in the head (H), tail (T), and rod domains (1A, 1B, 2A, 2B) of type II keratins (KRT1, KRT2, KRT5, KRT6) than of type I keratins (Table 1). However, the disease-causing variants observed in type I keratins (KRT9, KRT10, KRT13, KRT14, KRT16, KRT17) occur mostly in the rod domains (1A, 1B and 2B), except in the case of palmoplantar keratoderma. These data suggest that explanations of the phenotype (*i.e.,* the human disorder) caused by each of these mutations might range from disrupting the dimer, tetramer, and higher-order formation, to the IntFil-interacting interface with its dimeric partner.

**Table 1:**
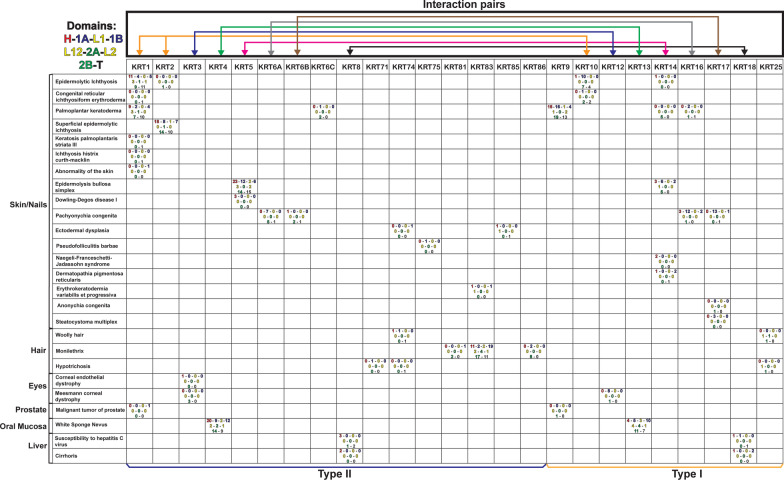
Distribution of 26 disease-causing variations in human keratin protein domains

Searching the ClinVar database for coding variations in 54 human type I and type II keratin genes revealed 26 variations classified as pathogenic (this includes susceptibility to hepatitis C virus). Names of the disorders caused by variations in keratin-coding sequences are shown in the left column. Keratin genes are listed in the row at top. Domain locations for pathogenic variants are designated as: Top row: Head (red); 1A (blue), L1 (gold), 1B (blue); Middle row: L12 (gold), 2A (green), L2 (gold); Bottom row: 2B (green), Tail (black). Keratin-interaction partners are indicated by colored lines as follows: KRT1, KRT2/KRT10 (orange), KRT3/KRT12 (blue), KRT4/KRT13 (green), KRT5/KRT14 (pink), KRT6A/KRT16 (grey), KRT6B/KRT17 (brown), KRT8/KRT18 (black). The number of variants in a keratin domain, associated with a given disorder, is displayed. Type II keratin proteins are shown at left and are indicated by a blue line along the bottom of the figure. Type I keratin proteins are exhibited at right and denoted by a gold line along the bottom of the figure

As indicated in Fig. 6, a large number of keratins are expressed in hair-nails-tongue. Therefore, it is expected that mutations in these keratin genes would lead to diseases of these tissues. Indeed, this is the case: defects in hair-nails-tongue keratin proteins—such as KRT25, KRT71, KRT74, KRT81, KRT83, KRT86—are involved in diseases of the hair and nails (Table 1). More specifically, variants in the *KRT74* and *KRT85* genes are associated with ectodermal dysplasia; *KRT25* and *KRT74* variants are associated with woolly hair; *KRT75* variants are associated with pseudo-folliculitis barbae; *KRT81, KRT83* and *KRT86* variants are associated with monilethrix; *KRT17* variants are associated with anonychia congenita; and variations in *KRT25, KRT71* and *KRT74* are associated with hypotrichosis (Table 1).

The GTEx database indicates that many keratin genes, including *KRT1*, *KRT10*, *KRT8, KRT18, KRT6A, KRT17*, *KRT5* and *KRT14,* are expressed in every tissue (Fig. 6). Interestingly, variants in keratin genes do not appear to cause disease in the vast majority of these tissues, aside from *KRT8/KRT18* (Table 1). *KRT8/KRT18* variations listed in the ClinVar database may be involved in nonalcoholic steatohepatitis (NASH), in oxidative stress to the liver, indirectly leading to cirrhosis [97, 98], and in increased formation of fibrosis during chronic hepatitis C infection [99].

These data, nevertheless, beg the question as to why alterations of ubiquitously expressed keratin genes (such as *KRT8/KRT18*) cause disease only in liver, and not in a multitude of other tissues. One possibility is that such changes disturb interactions between keratins and binding partner(s) (i.e., keratin-associated proteins)—rather than disrupting the integrity of the KRT8/KRT18 protein dimer itself; this hypothesis would make most sense if those mutated amino acids are located at the solvent-exposed molecular surface, or if the IntFil surface chemistry is altered.

Interactions of KRT6 with KRT16 or KRT17 are very intriguing. The evolutionary change of KRT1/KRT10 to KRT6/KRT17 in aquatic mammals (*Cetaceans*) suggests that KRT6/KRT17 might be associated with life in cold water [51], in which a thickened basal layer of epidermis would be beneficial (Fig. 5). In early studies on ridged skin of the human palm, KRT17 was found to be expressed in the basal layer of the primary epidermal ridge, whereas KRT16 expression occurs across the secondary epidermal ridge; this finding indicates that KRT17 plays a larger role than KRT16 in maintaining a high proliferation signal under high-stress conditions [86]. Accordingly, Coulombe and colleagues discovered that KRT17 has a high capacity to induce hyperproliferation signals—through the STAT3 and 14–3–3*σ* pathways [100, 101]. In contrast, KRT16 function appears to be more connected with KRT6 and maintenance of cellular mitochondrial organization [102], as well as with innate immunity [103].

Variations in the *KRT6A, KRT6B, KRT16* and *KRT17* genes are, in large part, associated with a rare disease related to thickening and abnormal shaped of fingernail and toenail, pachyonychia congenita (PC) (Table 1); while an absence of the *Krt16* gene in mouse causes thickening skin of palms and feet, palmoplantar keratoderma (PPK) [104]. PC-related variants are more frequently associated with type I genes *KRT16* (18 variants) and *KRT17* (15 variants) than with the type II genes *KRT6A* (13 variants), *KRT6B* (4 variants), and *KRT6C* (novariants). Furthermore, PC- and PPK-related variations primarily result in perturbation of the 1A and 2B domains of the keratin proteins, suggesting they distort either filament formation or how these keratins interact with other intracellular proteins [105].

Recent evidence suggests that KRT8 overexpression on the cell surface might enhance cell adhesion to the extracellular matrix—raising questions about involvement of KRT8 in cancer-cell-signaling pathways [106]. These studies suggest that IntFils may be potential targets for future therapeutics in prevention of viral infection and cancer treatment. Non-keratin IntFils have also recently been implicated in many diseases—including COVID-19 infection and cancer-cell signaling. For example, the IntFil type III vimentin was found to be upregulated in human cells infected with SARS-CoV and is suspected to facilitate entry of the virus into host cells [107]. Furthermore, a recent article deposited in bioRxiv suggests that extracellular vimentin acts as a critical component of the SARS-CoV-2 spike protein-ACE2 complex and that antibodies against vimentin can prevent SARS-CoV-2 infections in vitro [108].

## Conclusions

Intermediate filaments (IntFils), and in particular keratins, have been a focus of researchers for well over 50 years. IntFils are critical in intracellular and extracellular support to create distinct cell-types, tissues, organs, appendages, and body shapes. Our understanding of these multi-functional cytoskeleton proteins has advanced dramatically with the development of new investigative technologies. With respect to posttranslational keratin filament assembly, we now know that discrete molecular interactions can regulate higher-order keratin structures (e.g., a knob-pocket tetramerization mechanism in the 1B domain of type II keratins).

Paralogs (genes created by duplication events which often lead to diverse functions)—that have expanded rapidly in evolutionary time such that they exist as a cluster within a segment of the same chromosome—have been termed ‘evolutionary blooms.’ By examining human, mouse, and zebrafish phylogenetic trees, we show that keratin type I and type II clusters exist in genomes of human and mouse but not fish. These conserved clusters have also been found in seven other mammals (chimpanzee, macaque, pig, dog, cat, cow, horse) currently registered in the Vertebrate Gene Nomenclature Committee (vertebrate.genenames.org). Screening 259 species and subspecies in 20 phyla of animals, from jellyfish to human, we identified keratin proteins that appear to have arisen, disappeared, and sometimes reappeared. Between ~ 380 and ~ 150 million years, dozens of new forms of type I and type II keratin proteins were rapidly recruited in creating new anatomical structures needed during the transition of sea animals to land animals.

Analysis of keratin evolution also suggests that the type II keratins experienced more selective pressure than the type I keratins throughout time and thus type II keratins likely played a greater role in speciation of the animal kingdom. Despite experiencing less selective pressure than type II keratins, type I keratins nonetheless were involved in diversification of species and sub-speciation. Ultimately, the evolution of keratins reflects the evolutionary history of the animal kingdom.

Despite having similar coiled-coil structural folds, keratin proteins exhibit distinct surface chemistries that enable unique, diverse roles for keratins in extra- and intra-cellular functions—critical during embryonic development and establishing basic human physiology (e.g., epidermal skin barrier integrity). This functional diversity is directly correlated with multiple human diseases that can occur when humans acquire new variants/mutations in keratin genes, resulting in defective assembly, or altered keratin protein function. It is apparent that IntFils are involved in the etiology and/or progression of rare skin diseases, cancer, and possibly even COVID-19.

Interestingly though, the range of diseases caused by mutations in keratins is narrower than would be expected—given the expansive expression patterns of keratins in all cell-types of the human body. This peculiarity suggests that redundancies may exist among keratins, and perhaps among other IntFils, that remain to be elucidated. It is anticipated that studies which leverage next-generation technologies [e.g., CRISPR/*Cas9*, artificial intelligence (AI), machine learning (ML), and deep learning (DL)] to investigate these mysteries will have enormous therapeutic potential by uncovering novel mechanisms by which keratins might be targeted.

## Methods

### Maximum likelihood phylogenetic inference

Sequences were aligned in MAFFT using the L-INS-I local pair methodology with 10,000 iterative alignment steps. Evolutionary models were determined using ModelFinder as implemented in IQTree, using Bayesian Information Criteria (BIC) to select the optimal model and gamma rate categories. Maximum Likelihood Phylogenetic trees were then constructed using the optimal model in IQTree; 10,000 Ultrafast Bootstrap permutations were performed to measure tree consistency. Due to the potential for model violations, each bootstrap tree was further optimized using a hill-climbing nearest neighbor interchange (NNI) protocol. Ultrafast Bootstrap Scores more closely resemble probabilistic measures than standard non-parametric bootstraps—but still should not be interpreted as strict probabilities of branching support.

### Bayesian inference of animal keratin phylogenies

Multiple sequence alignments were generated using the interactive Fast-Fourier Transform method in MAFFT, building the guide tree five times in the progressive stage with 10,000 refinement iteration cycles. Evolutionary relationships were estimated by Markov-chain Monte Carlo (MCMC) using MrBayes and an amino-acid-rate matrix averaged across 10 canonical distributional models. Each phylogenetic tree was inferred by two independent MCMC simulations lasting for 2.0 × 10^7^ iterations, sampling every 1000 generations in parallel using the BEAGLE library. Sufficient sampling of the posterior distributions of each parameter was evaluated—using effective sample size (ESS) values, with ESS values > 100 indicating adequate sampling of target parameters. Parallel-chain convergence was checked, using the within-chain and between-chain variance potential scale reduction factor (PSRF). Independent runs were assessed for convergence, and appropriate levels of burn-in visually, through visual inspection of the marginal posterior probabilities versus the generation state. The sampled posteriors from the two independent executions were then combined to generate a maximum clade-credibility tree—summarizing the posterior distribution of estimated evolutionary relationships and branch lengths.

### Tissue-specific expression

Median tissue-specific expression values for human keratin genes were retrieved from the Genotype-Tissue Expression (GTEx) database v8 [53] for all available human tissues. Only keratin genes with transcripts-per-million (TPM) counts of ≥ 0.1 were counted as “significantly expressed” in that tissue, whereas genes that failed to meet this criterion were classified as “not expressed” in that tissue. TPM counts were loaded into the Galaxy web platform [109], and the heatmap2 program within this platform was used to create heatmaps with the following options “–transform logarithm base 10 (value + 1), –scale by row, –cluster columns maximum distance and complete.”

## Supplementary Information

**Additional file 1**. Table containing genes included in the phylogenetic tree for Type I Keratins.

**Additional file 2**. Table containing genes included in the phylogenetic tree for Type II Keratins.

## Data Availability

All data used in this publication are publicly available at GTEx database v8 (https://gtexportal.org/home/), Zebrafish Information Network database (https://zfin.org/), Human Intermediate Filament database (http://www.interfil.org/), Universal Protein Knowledgebase (https://www.uniprot.org/), and Mouse Genome Informatics database (http://www.informatics.jax.org/).

## Abbreviations

*BFSP1*: Filensin
*BFSP2*: Phakinin
CYPs: Cytochrome P450 monooxygenases
GTEx: Genotype-Tissue Expression project
*IFFO1* & *2*: Intermediate filament family orphans 1 & 2
IntFil: Intermediate filament
KRT: Keratin
MCMC: Markov-chain Monte Carlo
MUPs: Mouse urinary proteins
PC: Pachyonychia congenita
PPK: Palmoplantar keratoderma
SCGBs: Human secretoglobins
TPM: Transcripts-per-million
ULF: Unit length filament
